# Spiny mice are primed but fail to regenerate volumetric skeletal muscle loss injuries

**DOI:** 10.1186/s13395-024-00358-y

**Published:** 2024-10-29

**Authors:** Mackenzie L. Davenport, Amaya Fong, Kaela N. Albury, C. Spencer Henley-Beasley, Elisabeth R. Barton, Malcolm Maden, Maurice S. Swanson

**Affiliations:** 1 Department of Molecular Genetics and Microbiology, University of Florida, College of Medicine, Gainesville, FL 32610 USA; 2 Center for NeuroGenetics, University of Florida, Gainesville, FL 32610 USA; 3 UF Genetics Institute, University of Florida, Gainesville, FL 32610 USA; 4 Department of Biology, University of Florida, Gainesville, FL 32611 USA; 5 Department of Applied Physiology and Kinesiology, College of Health and Human Performance, University of Florida, Gainesville, FL USA; 6 Myology Institute, University of Florida, Gainesville, FL USA

**Keywords:** Muscle regeneration, Spiny mouse, *Acomys*, Volumetric muscle loss

## Abstract

**Background:**

In recent years, the African spiny mouse *Acomys cahirinus* has been shown to regenerate a remarkable array of severe internal and external injuries in the absence of a fibrotic response, including the ability to regenerate full-thickness skin excisions, ear punches, severe kidney injuries, and complete transection of the spinal cord. While skeletal muscle is highly regenerative in adult mammals, *Acomys* displays superior muscle regeneration properties compared with standard laboratory mice following several injuries, including serial cardiotoxin injections of skeletal muscle and volumetric muscle loss (VML) of the panniculus carnosus muscle following full-thickness excision injuries. VML is an extreme muscle injury defined as the irrecoverable ablation of muscle mass, most commonly resulting from combat injuries or surgical debridement. Barriers to the treatment of VML injury include early and prolonged inflammatory responses that promote fibrotic repair and the loss of structural and mechanical cues that promote muscle regeneration. While the regeneration of the panniculus carnosus in *Acomys* is impressive, its direct relevance to the study of VML in patients is less clear as this muscle has largely been lost in humans, and, while striated, is not a true skeletal muscle. We therefore sought to test the ability of *Acomys* to regenerate a skeletal muscle more commonly used in VML injury models.

**Methods:**

We performed two different VML injuries of the *Acomys* tibialis anterior muscle and compared the regenerative response to a standard laboratory mouse strain, *Mus* C57BL6/J.

**Results:**

Neither *Acomys* nor *Mus* recovered lost muscle mass or myofiber number within three months following VML injury, and *Acomys* also failed to recover force production better than *Mus*. In contrast, *Acomys* continued to express eMHC within the injured area even three months following injury, whereas *Mus* ceased expressing eMHC less than one-month post-injury, suggesting that *Acomys* muscle was primed, but failed, to regenerate.

**Conclusions:**

While the panniculus carnosus muscle in *Acomys* regenerates following VML injury in the context of full-thickness skin excision, this regenerative ability does not translate to regenerative repair of a skeletal muscle.

**Supplementary Information:**

The online version contains supplementary material available at 10.1186/s13395-024-00358-y.

## Background

Skeletal muscle, through the function of resident muscle stem cells or satellite cells (SCs), is a highly regenerative tissue which undergoes perpetual regeneration throughout life in response to injury [1–3]. Early in the response to injury following necrosis of the damaged tissue, signals from the innate immune system lead to satellite cell activation. From their normally quiescent state adjacent to the myofiber membrane, SCs undergo asymmetric division to generate differentiating myogenic precursors, or myoblasts, as well as to replenish the SC pool. In coordination with other resident cell populations to promote remodeling and repair, myoblasts fuse with each other or existing myofiber membranes to restore the muscle to its pre-injury state [1–3]. However, certain injuries, such as volumetric muscle loss (VML) due to ablation of significant muscle tissue, exceed the endogenous regenerative capabilities of muscle [3, 4]. This type of injury typically results in loss of muscle mass with frequent fibrotic and fatty replacement of the muscle tissue leading to severely compromised function.

There are few treatment options for VML injuries and most research into improving patient outcomes remains at the preclinical stage. The leading hypothesis for regenerative failure following VML injury is that loss of SCs, extracellular matrix (ECM), and connective tissue results in loss of the structural and mechanical cues for proper regeneration [4, 5]. This is evidenced by the remarkable recovery and regeneration of muscle following myotoxin injury in which the muscle fibers are destroyed while the ECM remains intact, in contrast to VML injuries where the ECM is lost and minimal regeneration occurs [4]. Most research efforts for VML have thus focused on the development of biomaterials and scaffolds with and without cell transplantation to promote muscle restoration [5, 6]. Unfortunately, these methods still result in functional deficits and incomplete recovery of muscle mass, especially when transitioned to larger animal models. There are also translational feasibility concerns with these approaches regarding the expense and complexity of manufacturing [5].

In contrast to engineering approaches to regenerate or repair skeletal muscle, another strategy is to investigate the molecular mechanisms promoting regeneration in other species with greater regenerative abilities than humans. For example, the MRL/Mpj mouse strain regenerates 2 mm ear punch wounds [7, 8]. Since this original observation, MRL/Mpj has demonstrated improved healing in a handful of other injuries [9], although it still produces a fibrotic scar in response to skin wounds [10, 11]. MRL/Mpj displays greater myofiber regeneration and reduced fibrosis compared to DBA/2J in a model of limb girdle muscular dystrophy [12]; however, the DBA/2J background intensifies the muscular dystrophy phenotypes compared to the C57BL/6, CD1, and 129/SVEMS + /J backgrounds [13]. The same is also true of DBA/2J compared to C57BL/10 in the Duchenne muscular dystrophy mouse model, *mdx* [14–18]. Recently Norris et al. reported an in-depth analysis comparing multiple mouse strains and different injury models, which highlighted regenerative differences in skeletal muscle both in response to injury type and strain background [19]. Thus, mouse strains fall along a continuum of regenerative ability, possibly with MRL/Mpj as the peak. However, following VML injury, MRL/Mpj develops more adipocytes, similar amounts of fibrosis, and no improved grip strength compared to C57BL/6 [20].

In contrast to mice and other mammals, many amphibians are capable of complete regeneration of many tissues including following autotomy of the tail, while the axolotl *Ambystoma mexicanum* stands perhaps as the most well-recognized example of regenerative capacity in the animal kingdom, capable of regenerating whole limbs following surgical amputation [21]. Notably, axolotls have also been shown to regenerate muscle following VML injuries, although they fall short of regenerating the total excision of a single muscle potentially due to failure to recognize the injury [22]. Such regenerative capacity was long thought absent from adult mammals until the discovery of several species of African spiny mouse (*Acomys kempi*, *Acomys percivali*, and *Acomys cahirinus*) [23]. In recent years, much research has focused on the improved regenerative capabilities of *Acomys cahirinus* (hereafter referred to as *Acomys*). *Acomys* has been shown to regenerate full thickness skin excision injuries [23–25] and burns [26], ear punches [23, 27–29], hemi-crush [30] and complete transection spinal cord injuries [31], myocardial infarctions [32–34], and severe obstructive and ischemic kidney injuries [35]. In addition to the remarkable regeneration of these internal and external injuries, *Acomys* is characterized by superior skeletal muscle regeneration compared to *Mus* following serial rounds of myotoxin injection, whereby *Mus* eventually develop fatty infiltration of the muscle while *Acomys* continue to regenerate their muscles with high fidelity [36]. Perhaps the most relevant injury to the study of VML, however, is the full thickness skin excision injury, where in addition to the scarless regeneration of the epidermis, dermis, hair follicles, erector pili smooth muscles, and sebaceous glands, the thin layer of striated muscle under the skin, the panniculus carnosus (PC), has also been shown to regenerate [24, 25]. While the regeneration of this injury is impressive and can be compared to VML as a muscle ablation injury, the direct comparison to VML injury in humans or other common animal models of VML injury is less clear. This is at least partially due to the PC not being a true skeletal muscle as it is not attached to the skeleton. Also, while the PC exists in many mammals and functions in skin twitching, it is thought to have been largely lost and is vestigial in humans, still present at only a few anatomical locations in the body [37]. We thereby sought to test the ability of *Acomys* to regenerate VML injuries in a limb skeletal muscle that is preserved across species, the tibialis anterior (TA) muscle.

## Methods

### Animals

Male and female C57BL6/J (Jackson Labs) 14-week-old mice and 16–18-week-old male and female *Acomys cahirinus*, obtained from the breeding colony housed at the University of Florida, were used for all studies.

### Experimental procedures

All procedures involving experimental animals were approved by the Institutional Animal Care and Use Committee at the University of Florida (protocol number 202107707 for *Acomys cahirinus* and 20203677 for *M. musculus*). For tibialis anterior (TA) VML experiments, animals were anaesthetized with isoflurane, administered Buprenorphine SR (1 mg/kg) subcutaneously, the left hindleg shaved and a small incision made in the skin over the superior surface of the lower hindlimb to expose the TA body, and the fascia overlying the TA was dissected away. For biopsy punch VML injuries, a small metal spatula was inserted under the TA muscle and a 3 mm (C57BL6/J females), 3.5 mm (C57BL6/J males), or 4 mm (*Acomys cahirinus*) biopsy punch was used to create a uniform injury in the TA midbelly followed by skin suturing. For VML trough injuries, a sterile scalpel was used to make parallel ~ 2 mm deep cuts ~ 2 mm apart and ~ 5-6 mm long creating 20–30% ablation of the TA muscle and incisions were also sutured. The wet weight of the excised muscle was measured: C57BL6/J female 9.6 ± 1.5 mg, C57BL6/J male 13.1 ± 1.5 mg, *Acomys cahirinus* 17.7 ± 2.2 mg. Animals with injuries smaller than 20% or larger than 30%, calculated based on additive weight of the excised and remaining TA upon collection or compared to the uninjured TA, were excluded from analysis.

### Histology

Muscles were embedded in OCT, frozen in liquid nitrogen cooled isopentane, and stored at -80 °C until further analysis. 10 μm thick sections were stained for hematoxylin (Polysciences 24,244) and eosin (Polysciences 09859) (H&E). For picrosirius red staining, slides were fixed in 4% PFA for 45 min before following standard protocols. Picrosirius Red Solution contained 0.1% Direct Red 80 in saturated Picric Acid. Slides were scanned at 20X using a Motic Slide scanner and .tif files exported using Leica Aperio ImageScope software.

### Immunofluorescence

Slides were blocked for 45 min in 5% donkey serum and 0.3% TritonX-100 in PBS. For mouse primary antibodies donkey anti-mouse Fab fragments (Jackson Immunoresearch # 715–007-003) were added to the blocking buffer (1:50). Primary antibodies were incubated for 3 h at room temperature in blocking solution, followed by 3 washes with PBS-T, and 1 h incubations in secondary antibody. Slides were mounted with FluorSave. Antibodies: Laminin (Sigma L9393, 1:1000), eMHC/*MYH3* (DSHB F1.652, 1:40), Perilipin (Cell Signaling 9349S, 1:1000), Phalloidin (Invitrogen A12380, 1:250), PDGFRα (R&D AF1062, 1:250), CD206/MMR (R&D AF2535, 1:40), Ki67 (Invitrogen SolA15 14–5698-82, 1:1000), Pax7 (DSHB, 1:40), MyoD (ActiveMotif 5F11 39992, 1:200), DAPI (Sigma D9542, 1:25,000). Images were acquired with either a Zeiss Axioscan 7 or Echo Revolution automated microscope.

### Image quantification

For total myofiber number quantification and myofiber size distribution analysis, whole TA cross-section images were segmented by Cellpose [38] using the modified GoogleColab script written by Ariel Waisman followed by myofiber identification using the FIJI plugin LabelstoROIs [39]. Misidentified myofibers were manually corrected. Centralized nuclei and eMHC + fibers were counted manually for one cross-section per TA and represented as a percent of the total myofibers per TA cross-section. Adipocytes were also counted manually and due to the high variation in number of adipocytes per section, at least 3 sections > 100 µm apart were counted and averaged. FAPs were quantified by percent area PDGFRα + using color segmentation within a 1355µm^2^ region of injured area of one cross-section per TA. Fibrosis was quantified by %red / (%red + %yellow) picrosirius red staining using the PSR_quantify.ijm macro for FIJI [40, 41].

### Muscle functional assessment

#### In vivo torque

Mice were anaesthetized with isoflurane, then transferred to a heated platform while maintaining isoflurane inhalation by nose cone. The foot was strapped onto a footplate in series with a torque transducer using surgical tape. The knee was stabilized by a clamp on the platform. The dorsiflexor muscles were stimulated via the peroneal nerve with bipolar transcutaneous electrodes. Twitches elicited by single 0.2 ms pulses were used to optimize electrode placement, current amplitude, and the optimal tibiotarsal angle for the dorsiflexors (typically 110º of plantarflexion). Next, 150 Hz pulses within a 500 ms pulse train were used to determine optimal torque.

#### In situ force

Mice were anaesthetized with isoflurane, then the distal tendons of one tibialis anterior (TA) muscle at a time were dissected free from surrounding tissue and individually tied with 5.0 braided surgical silk and subsequently cut at their most distal ends. The sciatic nerve was exposed and all its branches cut except for the common peroneal nerve (CPN), which innervates the TA muscle. The mouse was then placed on a heating pad to maintain body temperature at 37 °C. The foot was secured to a platform and the knee immobilized using a clamp. The TA tendon was attached to a force transducer (Aurora Scientific) to record muscle twitch forces. Isometric twitch contractions were elicited by stimulating the distal part of the nerve via bipolar stainless steel electrodes using square wave pulses of 0.02 ms. The stimulation voltage and subsequently the length of the muscle were adjusted to produce the maximum isometric twitch force. The stimulus amplitude was then set to 0 V and manually increased over a range of 10 V, which results in discrete increments in twitch force due to the successive recruitment of motor units. This procedure was repeated until there was no further increase in force, indicating that all motor units were recruited. Force output was recorded using an analogue to digital converter interfaced with a computer running the appropriate software (Dynamic Muscle Control version 5.5).

## Results

### Compared to *Mus*, the *Acomys* TA is larger and muscle size is more consistent between the sexes

**Fig. 1:**
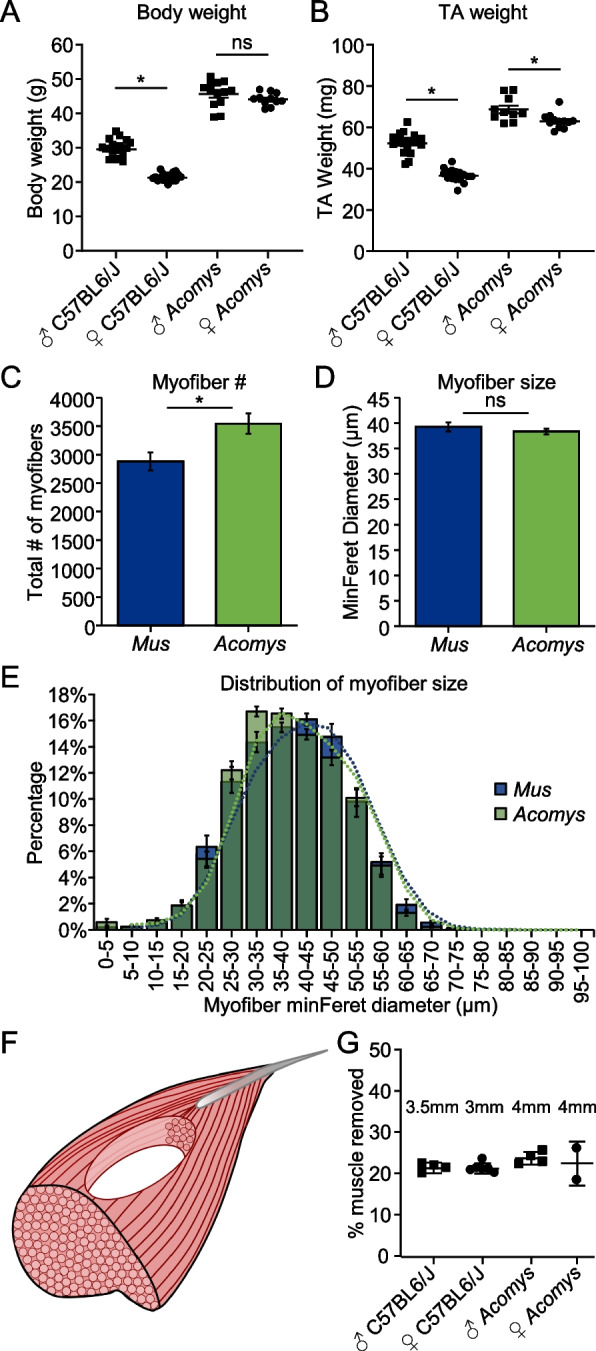
Modeling VML injuries across species. **A** Body weight (g) differences between male and female C57BL6/J *Mus* and *Acomys* (error bars ± SEM: *, *p* < 0.05, Student’s t-test). *n* = 20 male *Mus*, *n* = 17 female *Mus*, *n* = 13 male *Acomys*, *n* = 11 female *Acomys*. **B** TA weight (mg) differences between male and female C57BL6/J *Mus* and *Acomys* (error bars ± SEM: *, *p* < 0.05, Student’s t-test). **C** Total number of myofibers per TA cross-section in *Mus* and *Acomys* (error bars ± SEM: *, *p* < 0.05, Student’s t-test). *n* = 12 *Mus*, *n* = 9 *Acomys*. **D** Average MinFeret diameter of myofibers per TA cross-section in *Mus* and *Acomys* (error bars ± SEM: *, *p* < 0.05, Student’s t-test). *n* = 12 *Mus*, *n* = 9 *Acomys*. **E** Myofiber size distribution by MinFeret diameter in *Mus* and *Acomys* (error bars ± SEM). Bars are overlapping. **F** Model diagram of biopsy punch injury in TA. **G** Mean % muscle removed by wet mass of *Mus* and *Acomys* TA muscles using indicated biopsy punch sizes (error bars ± SD: *, *p* < 0.05, One-way ANOVA with Tukey’s multiple comparisons test)

While many comparisons of skeletal muscle between *Acomys* and *Mus* have already been reported, including weight differences in the EDL, soleus, and TA muscles, myofiber number and length differences in the EDL and soleus, and fiber type differences in the EDL, soleus, and TA [36], we sought to further characterize differences in the TA muscles between the species so we could best determine how to consistently create muscle injuries in the two species. Additionally, it is well known that female *Mus* are smaller and have respectively smaller muscles compared to male *Mus*. This has not previously been clear for *Acomys*, and due to challenges in *Acomys* breeding (smaller litter size, longer gestation, longer time to sexual maturity), we sought to use both sexes for our studies. We determined that on average, female *Acomys* are not significantly smaller by body weight than male *Acomys* at ~ 4 months of age, compared to female C57BL6/J (*Mus*) mice which are significantly smaller than their male counterparts at 14 weeks of age (Fig. 1A). Similarly, while the TA muscles of female *Acomys* are statistically smaller than the TA muscles of male *Acomys*, the average difference in mass is less than 10%. This is compared to *Mus* where female TA muscles are 30% smaller than males (Fig. 1B). Furthermore, the total number of myofibers and the minFeret diameter of TA myofibers were not different between male and female *Acomys*, as opposed to *Mus* where females have a similar number but smaller myofibers compared to males (Figure S1A-F). We do not observe overt differences in ECM or fat content to explain the < 10% weight difference in male and female *Acomys* TAs. We therefore combined data from male and female *Acomys* for our studies. *Acomys* are, however, ~ 35% bigger by body weight than male *Mus* and ~ 50% bigger than female *Mus*, and their TAs are 25%—40% heavier, respectively (Fig. 1A, B). As has been reported for the EDL and soleus, we determined that the size difference in *Mus* and *Acomys* TA muscles is due to a greater number of myofibers in the *Acomys* TA (Fig. 1C), while myofiber size is similar between the species (Fig. 1D, E). *Acomys* myofiber size distribution falls in between that of male and female *Mus* (Figure S1F). The *Acomys* TA is also longer than the *Mus* TA.

One common model of volumetric muscle loss injury in rodents is a biopsy punch of the TA muscle (Fig. 1F). To create similar size injuries between the species, we tested a variety of biopsy punch sizes in the TA of each species and determined that a 3.5 mm punch in male *Mus*, a 3.0 mm punch in female *Mus*, and 4.0 mm punch in both sexes of *Acomys* removed 20–25% of the TA muscle (Fig. 1G).

### *Acomys* and *Mus* respond similarly to VML biopsy punch injuries but eMHC expression remains elevated in *Acomys*

**Fig. 2:**
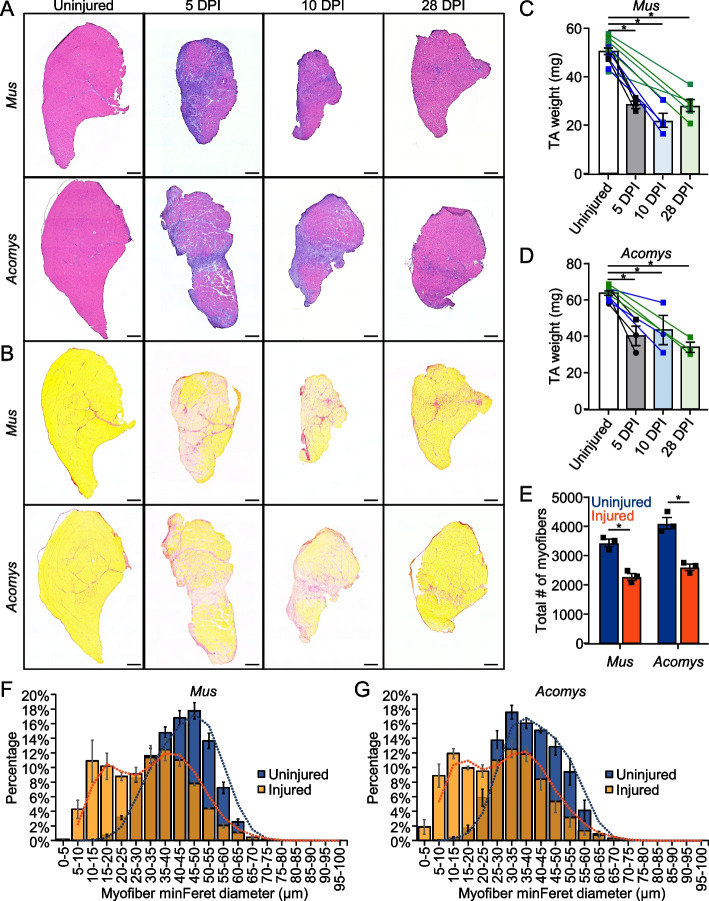
*Acomys* and *Mus* display similar injury responses to VML biopsy punch. **A**, **B** (**A**) H&E stained and (**B**) picrosirius red stained cross-sections of uninjured, 5 days post biopsy punch injury (DPI), 10 DPI, and 28 DPI *Mus* and *Acomys* TA muscles. Scale bar = 400 μM. **C**, **D** Mean TA weights 5, 10, and 28 DPI in (**C**) *Mus* and (**D**) *Acomys* (error bars ± SEM: *, *p* < 0.05, One-way ANOVA with Dunnett’s multiple comparisons test). Lines indicate contralateral TA muscles of the same animal. Squares indicate males. Circles indicate females. **E** Total number of myofibers per TA cross-Sect. 28 DPI in *Mus* and *Acomys* (error bars ± SEM: *, *p* < 0.05, 2-way ANOVA). **F**, **G** Myofiber size distribution by MinFeret diameter 28 DPI in (**F**) *Mus* and (**G**) *Acomys* (error bars ± SEM). Bars are overlapping

**Fig. 3:**
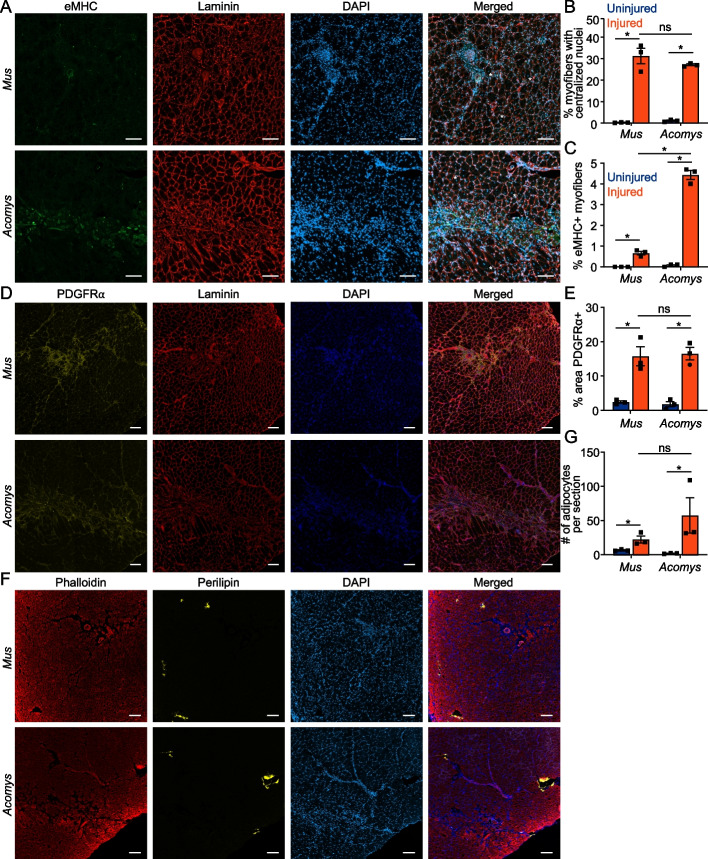
eMHC expression persists in *Acomys* 28 days following VML biopsy punch. **A** Immunofluorescence of eMHC, Laminin, and DAPI 28 DPI in *Mus* and *Acomys*. Scale bar = 100 μM. *Indicates centrally nucleated myofibers. **B** Quantification of percent myofibers with centralized nuclei per cross-Sect. 28 DPI in *Mus* and *Acomys* (error bars ± SEM: *, *p* < 0.05, 2-way ANOVA). **C** Quantification of percent eMHC + myofibers per cross-Sect. 28 DPI in *Mus* and *Acomys* (error bars ± SEM: *, *p* < 0.05, 2-way ANOVA). **D** Immunofluorescence of PDGFRα, Laminin, and DAPI 28 DPI in *Mus* and *Acomys*. Scale bar = 100 μM. **E** Quantification of FAPs by percent area PDGFRα + within a 1355 µm.^2^ area of the wound center (error bars ± SEM: *, *p* < 0.05, 2-way ANOVA). **F** Immunofluorescence of Phalloidin, Perilipin, and DAPI 28 DPI in *Mus* and *Acomys*. Scale bar = 100 μM. **G** Average number of adipocytes per TA cross-Sect. 28 DPI in *Mus* and *Acomys* (error bars ± SEM: *, *p* < 0.05, 2-way ANOVA)

We initially assessed multiple timepoints following injury to determine if *Mus* and *Acomys* displayed similar wound healing trajectories. H&E staining and picrosirius red staining to assess fibrosis of biopsy punch VML injuries 5-, 10-, and 28-days post injury (DPI) indicated similar progression between the species (Fig. 2A, B, S2A-C). Both species appeared to have early inflammatory and fibrotic responses to injury. Immunofluorescence of CD206 + macrophages indicated similar inflammatory profiles between the species at all timepoints where macrophages were abundant both within the injured area and throughout the interstitial spaces of the injured muscle (Figure S3). Neither species recovered mass of the injured TA by 28DPI (Fig. 2C, D). Similarly, both species showed a similar decrease in myofiber number 28DPI (Fig. 2E), with a shift toward smaller myofibers (Fig. 2F, G). Both species also developed a similar percentage of centronucleated myofibers (Fig. 3A, B). In contrast, fibers positive for eMHC were abundant within the center of the wounded area in *Acomys*, while only a few sporadic eMHC + fibers were present in *Mus* TA 28DPI (Fig. 3A, C, S4). This is despite an initially greater increase in eMHC + myofibers in *Mus* at 5DPI which dramatically decreased by 10DPI (Figure S5A-C). The percentage of myofibers expressing eMHC did not differ significantly between 5 and 10DPI in *Acomys*. Since *Acomys* muscle appeared to have a more persistent regenerative response, we asked whether the number of fibroadipogenic progenitors (FAPs), another cell type critical for muscle regeneration [42–44], differed between the species. However, we did not detect a difference in FAPs between *Mus* and *Acomys* 28DPI (Fig. 3D, E, S6), nor did we detect fewer adipocytes in *Acomys* as we hypothesized based on published serial cardiotoxin injuries (Fig. 3F, G, S7) [36]. Of note, there was considerable variability among animals of both species in the number of adipocytes observed following injury (Figure S7).

### Persistent eMHC expression in *Acomys* following a less severe VML trough injury

**Fig. 4:**
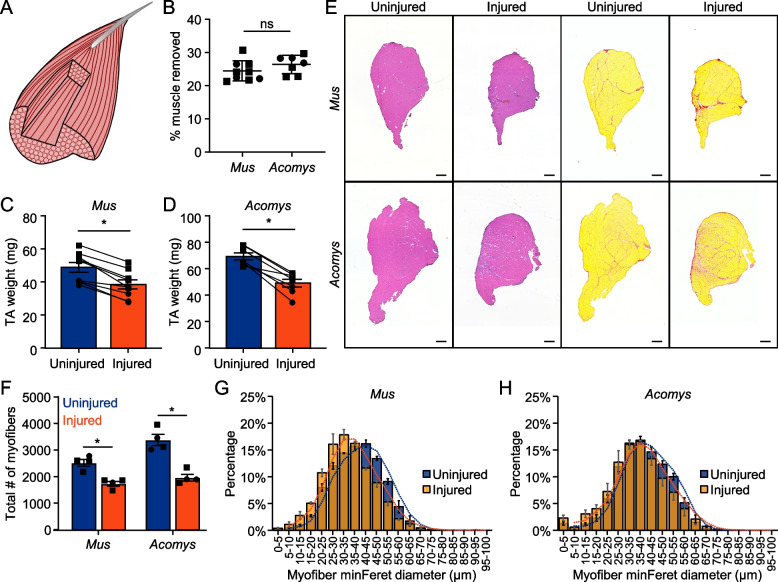
*Acomys* and *Mus* display similar injury responses to VML trough injury. **A** Model diagram of TA trough injury. **B** Mean % muscle removed by wet mass of *Mus* and *Acomys* TA muscles following trough injuries (error bars ± SD: *, *p* < 0.05, Student’s t-test). *n* = 9 *Mus*, *n* = 7 *Acomys*. **C**, **D** Mean TA weights 90 DPI in (**C**) *Mus* and (**D**) *Acomys* (error bars ± SEM: *, *p* < 0.05, Student’s t-test). **E** H&E and picrosirius red stained cross-sections of *Mus* and *Acomys* TA muscles 90 days post VML trough injury. Scale bar = 400 μM. **F** Total number of myofibers per TA cross-Sect. 90 DPI in *Mus* and *Acomys* (error bars ± SEM: *, *p* < 0.05, 2-way ANOVA). **G**, **H** Myofiber size distribution by MinFeret diameter 90 DPI in (**G**) *Mus* and (**H**) *Acomys* (error bars ± SEM)

**Fig. 5:**
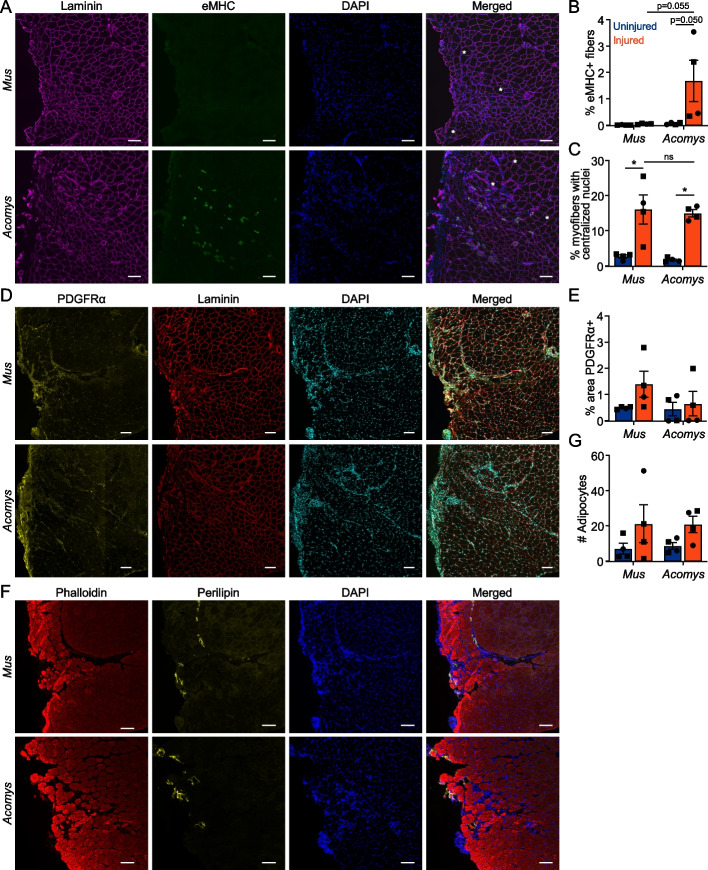
eMHC expression persists in *Acomys* 90 days following VML trough injury. **A** Immunofluorescence of eMHC, Laminin, and DAPI 90 DPI in *Mus* and *Acomys*. Scale bar = 100 μM. *Indicates centrally nucleated myofibers. **B** Quantification of percent eMHC + myofibers per cross-Sect. 90 DPI in *Mus* and *Acomys* (error bars ± SEM: *, *p* < 0.05, 2-way ANOVA). **C** Quantification of percent myofibers with centralized nuclei per cross-Sect. 90 DPI in *Mus* and *Acomys* (error bars ± SEM: *, *p* < 0.05, 2-way ANOVA). **D** Immunofluorescence of PDGFRα, Laminin, and DAPI 90 DPI in *Mus* and *Acomys*. Scale bar = 100 μM. **E** Quantification of FAPs by percent area PDGFRα + within a 1355 µm.^2^ area of the wound center (error bars ± SEM: *, *p* < 0.05, 2-way ANOVA). **F** Immunofluorescence of Phalloidin, Perilipin, and DAPI 90 DPI in *Mus* and *Acomys*. Scale bar = 100 μM. **G** Average number of adipocytes per TA cross-Sect. 90 DPI in *Mus* and *Acomys* (error bars ± SEM: *, *p* < 0.05, 2-way ANOVA)

Because we did not detect overt differences in the TA muscle’s ability to regenerate biopsy punch VML wounds between *Mus* and *Acomys* at 28DPI, we tested a less severe VML injury. The biopsy punch sizes previously described create an initial injury which removes 20–25% of the TA muscle, but in *Mus* these defects result in 55.8% ± 2.5% and 45.4% ± 10.4% muscle remaining at 5 and 10 DPI, respectively (Fig. 2C). To produce a less severe injury, a scalpel was used to create a trough-like VML injury in the TA muscle which created 20–30% deficit, but which resulted in less immediate myofiber loss as the myofibers were not severed all the way through the TA (Fig. 4A, B). At 7 DPI, the muscle mass remaining was 64.8% ± 7.7% (data not shown). We also examined the TA muscles following this injury at 90DPI (~ 12 weeks or ~ 3 months) instead of 28DPI to allow *Acomys* more time to regenerate given the observed persistent eMHC expression at 28DPI following biopsy punch. At 90DPI, neither species recovered mass of the TA (Fig. 4C, D) and both species still appeared overtly injured (Fig. 4E, S8). Neither species recovered the number of myofibers, and both species still had a greater percentage of smaller myofibers, although the size distribution of myofibers had returned to a more normal distribution in *Acomys* compared to *Mus* (Fig. 4F-H). As with the biopsy punch injury, *Acomys* TA muscles still displayed persistent expression of eMHC + myofibers even three months following injury, while *Mus* lacked elevated eMHC expression (Fig. 5A-B, S9). The percent of centronucleated myofibers (Fig. 5C), as well as the number of FAPs (Fig. 5D, E, S10) and adipocytes remained similar between the species (Fig. 5F, G, S11). To determine if the eMHC + myofibers in *Acomys* at 90DPI were associated with activated or proliferating satellite cells, TA sections were stained with Pax7, MyoD, and Ki67; however, no increase in Pax7 + /MyoD + cells was observed compared to uninjured muscle (data not shown). Only one animal (25%) from the *Acomys* group showed an increase in the number of Ki67 + cells compared to *Mus* at 90DPI (Figure S12).

### *Acomys* muscle function does not fully recover following VML injury

**Fig. 6:**
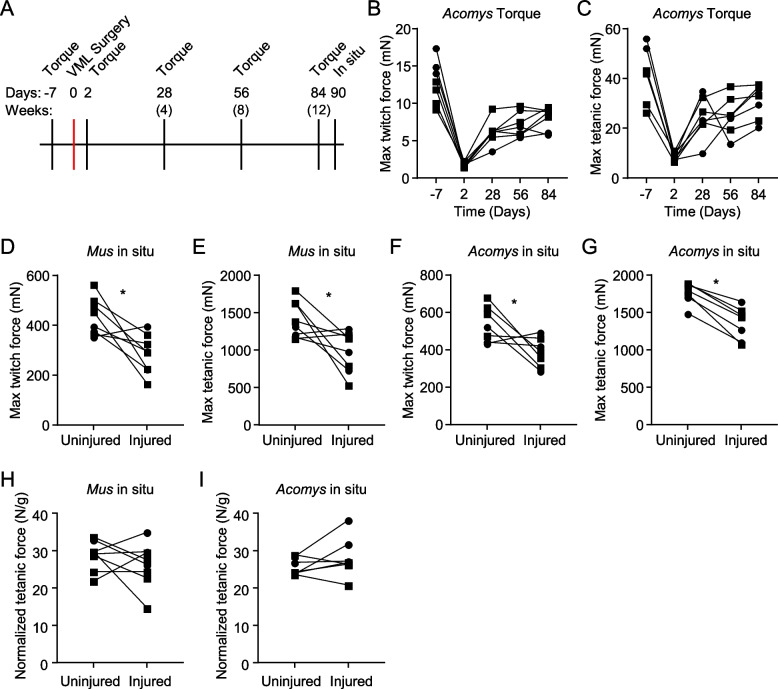
*Acomys* does not functionally recover 90 days following VML trough injury. **A** Experimental diagram indicating timepoints of in vivo torque measurements, surgery, and final in situ measurements. **B**, **C** In vivo torque measurements in *Acomys* pre- and post-VML trough injury (**B**) Maximum twitch force, (**C**) Maximum tetanic force. **D**-**I** In situ force production measurements of uninjured and injured TA muscles in (**D**, **E**) *Mus* and (**F**, **G**) *Acomys* 90 DPI (*, *p* < 0.05, paired t-test). **D**, **F** Maximum twitch force. **E**, **G** Maximum tetanic force. **H**, **I** In situ tetanic force production normalized to TA mass

To assess whether *Acomys* might recover force production post-VML as well as assess if there was any improvement over time, we performed a series of in vivo torque measurements before and following trough VML injury (Fig. 6A). As expected, there was a strong decrease in both maximum twitch (85% ± 5.0%) and maximum tetanic (79% ± 7.3%) force 2 DPI (Fig. 6B, C). At 28DPI, *Acomys* maximum twitch force had improved to 52% ± 24.9% compared to baseline, and maximum tetanic force had improved to 63% ± 28.5% compared to baseline. While some individual animals continued to improve at 56- and 84-DPI, force production largely plateaued after 28DPI. By 84DPI, *Acomys* had recovered 69% ± 23.9% maximum twitch force compared to baseline and 79% ± 27.0% maximum tetanic force (Fig. 6B, C). Of note, in vivo torque measurements measure the maximal force of dorsiflexion, and thus in addition to force produced by the TA, also measure some force input from the EDL and EHL. To measure only the force produced by the TA as well as compare the *Acomys* response in force recovery to *Mus*, we performed in situ mechanics on the TA muscles of both species ~ 90 DPI (3 months). *Mus* and *Acomys* showed similar deficits in force production 90DPI by both maximum twitch and maximum tetanic force, with *Mus* recovering an average of 68.4% maximum twitch and 72.7% maximum tetanic force and *Acomys* recovering 75.4% maximum twitch and 77.0% maximum tetanic force on average (Fig. 6D-G). When these forces are normalized to the muscle remaining, *Acomys* fairs slightly better than *Mus* producing on average 109.9% ± 18.6% normalized tetanic force compared to their contralateral control limb with 71% of animals generating normalized forces in their injured limb equal to or greater than their contralateral control limb. This is compared to *Mus* which generates an average 93.1% ± 26.5% normalized tetanic force compared to their contralateral control limb with only 50% of animals generating normalized forces in their injured limb equal to or greater than their contralateral control (Fig. 6H, I). However, these differences do not reach statistical significance (*p* = 0.09, student’s t-test).

## Discussion

VML injuries result in loss of recoverable muscle mass, fibrosis, and severe functional impairment. *Acomys* was recently shown to regenerate full-thickness skin excision and thermal burn injuries [24–26], which are relevant to the study of VML as the panniculus carnosus (PC) muscle under the skin regenerates. Therefore, we tested if *Acomys* could also regenerate VML injuries of a skeletal muscle commonly used to model VML injury in rodents. Despite the remarkable regeneration of the PC muscle following full-thickness skin excision or burn injury in *Acomys*, the TA muscle of *Acomys* did not regenerate significantly better than the *Mus* TA following either of two VML injury models. Neither species recovered muscle mass, myofiber number, nor function following VML, and both species developed similar fibrotic and fatty infiltration of the muscle. While it might not be surprising that an adult mammal cannot regenerate a VML injury, it is quite surprising from an *Acomys* centric viewpoint since *Acomys* has been shown to regenerate acute obstructive and ischemic kidney injuries, fully regenerating nephron structure and organ function [35]; myocardial structure and cardiac function following acute and permanent left anterior descending coronary artery ligation [32–34]; and hemi-crush and complete transection spinal cord injuries, regenerating axons of multiple tracts, recovering bladder voiding, and regaining weight bearing support, plantar stepping, and limb coordination [30, 31].

The leading hypotheses regarding how highly regenerative skeletal muscle fails to regenerate following VML injury center around prolonged inflammatory responses, the deposition of a fibrotic zone within the wound that prevents the formation of new myofibers, and loss of the structural and mechanical cues for proper regeneration [3, 45, 46]. As a result, many studies have attempted to improve VML outcomes by modulating immune and fibrotic signaling [47–51]. Critically, almost every injury reported in which *Acomys* has an improved ability to regenerate has noted a lack of fibrotic response in this species and an altered, dampened inflammatory response [24–26, 28–35, 52–55]. This is one of the leading hypotheses for how *Acomys* regenerates many traditionally ‘non-regenerative’ injuries. We had thus hypothesized that *Acomys* would also not mount a fibrotic response to VML but were surprised to find similar amounts of fibrosis within the *Acomys* wounds compared to the *Mus* wounds using both VML models. We also observed similar inflammatory profiles of CD206 + macrophages across multiple timepoints following biopsy punch injury, indicating that the dampened immune response typical following injury in *Acomys* may not apply to VML wounds of the TA; however, CD206 + macrophages do not represent the total inflammatory response, and additional markers would need to be evaluated to definitively state this.

Despite the fibrotic response, *Acomys* was able to form new eMHC + myofibers within the injured fibrotic regions, surpassing one barrier to VML regeneration that *Mus* cannot. We did not observe eMHC + myofibers in the fibrotic regions of the *Mus* injuries at 28 or 90DPI, which concurs with previous reports of the absence of SCs from the defect zone of *Mus* VML injuries [56]. It is important to note that *Mus* does exhibit eMHC + myofibers at early timepoints following VML injury which quickly disappear, and both species develop centrally nucleated myofibers, suggesting that both species initially attempt to regenerate the injury but perhaps *Mus* fails faster. Myofiber size distribution also appeared more normal in *Acomys* 90DPI than *Mus* despite the increased number of eMHC + myofibers. The eMHC + myofibers present at 90DPI were smaller fibers as is expected of new or regenerating myofibers, suggesting that the myofibers that had returned to normal size had shut off eMHC expression. The explanation for the persistent decrease in myofiber size without the presence of newly regenerating myofibers in *Mus* is less clear but perhaps represents the injured state of the muscle and a lack of remaining satellite cells to promote hypertrophy of existing or damaged fibers. The presence of eMHC + myofibers in the *Acomys* wounds unfortunately did not lead to improved functional recovery compared to *Mus*, highlighting new challenges facing the VML field, as perhaps the formation of new myofibers within the wounded area is insufficient for recovery. Perhaps, the eMHC + myofibers persisting in the *Acomys* wounds indicate that *Acomys* is primed to regenerate but requires further molecular cues to complete the regenerative process.

There are several potential caveats to our findings. First, the injuries performed in this study were unilateral, and while the animals generally begin ambulating immediately following cessation of anesthesia, they may have favored the injured limb, subjecting it to insufficient force or exercise. We chose to perform unilateral injuries so that we could control for TA size differences between animals and have intra-animal controls given notable inter-animal variability, but there is a concern that the uninjured limb may compensate, and thus bilateral injuries may prove insightful. This is relevant as it has been reported that bilateral VML injuries exhibit reduced functional deficits compared to unilateral injuries in rats [57]. Second, exercise such as voluntary wheel or treadmill running has been reported as beneficial for functional recovery following VML in other rodent models [58–60], and thus exercise might provide a further cue to trigger additional regeneration in *Acomys* which might exceed that in *Mus*. Although, *Acomys* generally exhibit increased cage behavior such as chasing, running, and jumping compared to *Mus*, and the feasibility of traditional rodent exercise regimens in *Acomys* remains to be determined.

The regeneration of large skin injuries in *Acomys* comprising the PC initially suggested that *Acomys* might not require the same structural and mechanical cues for successful muscle regeneration as other mammals. However, the PC is not injured in isolation in these injuries, and the epidermis and dermis regenerate before the PC thus potentially providing the structural and mechanical cues necessary for PC muscle regeneration which are not present in the TA VML injury. This raises the question of whether *Acomys* might better regenerate an open wound, prompting skin contracture and regeneration and thus the potential release of paracrine factors which might stimulate muscle regeneration. However, this scenario is unlikely for the TA since it is not intimately connected to the skin the way that the PC is. It is also possible that the general properties of the PC simply allow for complete regeneration. While the fiber type profile of the PC is actually quite similar to the TA [61], it does not attach to the skeleton and thus its function and the forces exerted on it are completely different. It has been suggested even in *Mus* that the PC is more regenerative than other adult muscles, maintaining a higher number of centrally nucleated myofibers and incorporating a high percentage of bone marrow derived cells (BMDCs) [61, 62]. Similar numbers of satellite cells per myonuclei have been reported between the TAs of *Mus* and *Acomys* [36], but little is known about the satellite cells of the PC in *Acomys*. The satellite cells of the PC in *Mus* have been shown to originate from the same Myf5 + , Pax3/Pax7 + progenitors as skeletal muscle satellite cells [63], and the same is assumed of *Acomys*. Additionally, BMDCs in *Mus* can repopulate the satellite cell niche within the PC, but they fail to contribute to myogenic differentiation in vitro [63]. It would thus be interesting to see whether there are differences in satellite cell activation in the *Acomys* PC or whether BMDCs contribute to the PC of *Acomys* as well and whether they have greater myogenic potential. Finally, the PC is an extremely thin layer of muscle, and there may be an injury threshold that the PC does not meet but TA VML injuries exceed. There have been several studies showing such a threshold effect of VML injury in more common rodent models, where rodents can regenerate small VML injuries up to a certain threshold beyond which fibrotic repair replaces the regenerative response [49, 50, 64].

## Conclusions

While spiny mice regenerate a remarkable array of severe injuries, there are limits to their regenerative success. *Acomys* displays superior muscle regenerative capacity compared to *Mus* following biopsy punch of the ear pinna, full-thickness skin excision injury, and TA cardiotoxin injury, and regenerates most injuries in the absence of a fibrotic scar. However, we show here that a fibrotic response occurs in *Acomys* following two different VML injuries. While the *Acomys* TA appears primed to regenerate following VML injury, it ultimately fails, highlighting the severity of this injury and the complications faced when trying to develop therapeutic strategies to treat VML.

## Supplementary Information

Supplementary Material 1.

## Data Availability

All data generated or analyzed during this study are included in this published article [and its supplementary information files].
