# Supplementary material for: Spiny mice are primed but fail to regenerate volumetric skeletal muscle loss injuries
Source: Skelet Muscle. 2024 Oct 29;14:26. doi: 10.1186/s13395-024-00358-y (PMC11520498; doi:10.1186/s13395-024-00358-y)
Supplement: Supplementary file 1 — Supplementary Material 1. [file 13395_2024_358_MOESM1_ESM.pdf]

Supplemental Figure 1: **Davenport et al.**

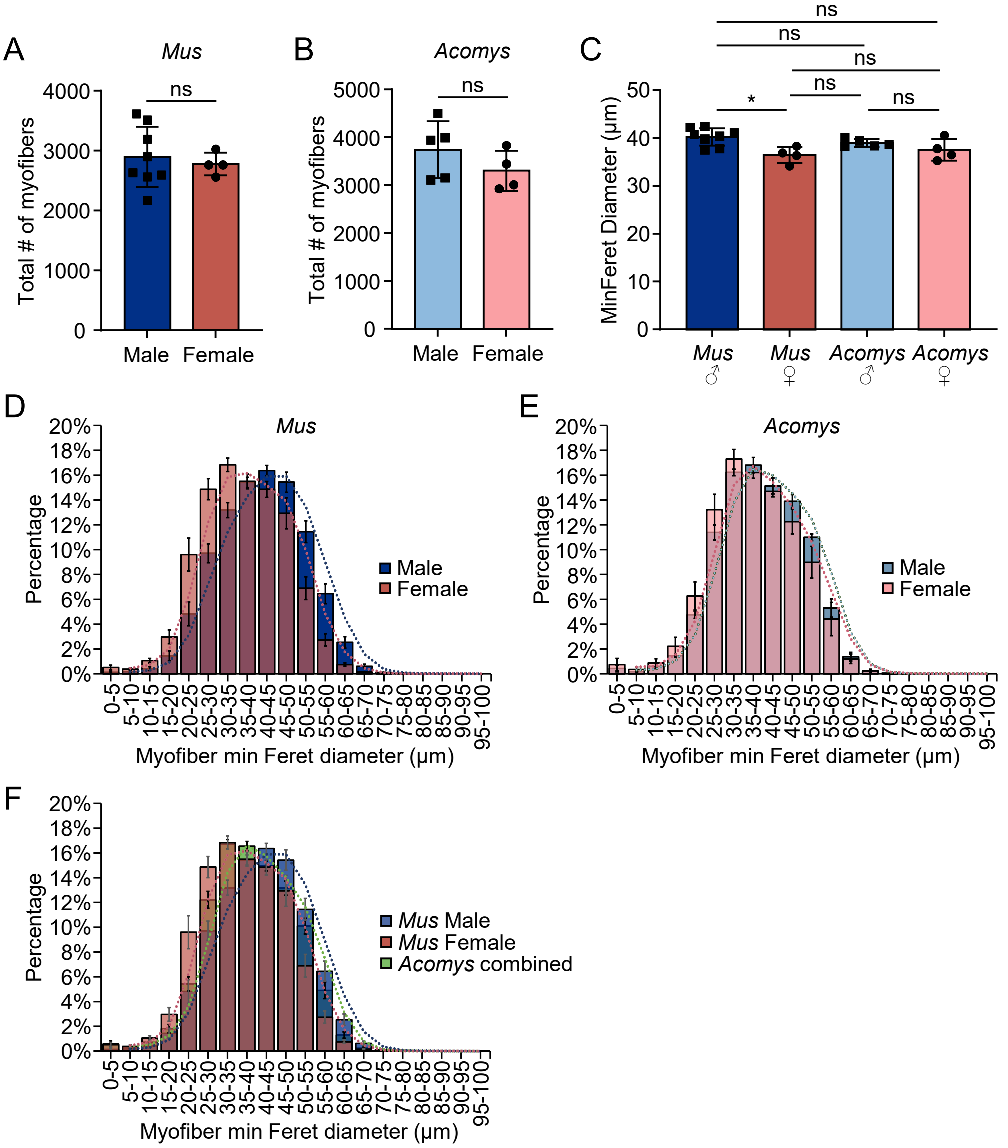

Figure S1. Sex differences in TA muscle between species. A-B) Total number of myofibers per TA cross-section in male and female *Mus* (A) and *Acomys* (B) (error bars ± SD: *, $p < 0.05$, Student's t-test). C) Average MinFeret diameter of myofibers per TA cross-section in male and female *Mus* and *Acomys* (error bars ± SD: *, $p < 0.05$, One-way ANOVA with Tukey's multiple comparison test). D-F) Myofiber size distribution by MinFeret diameter in male and female *Mus* (D) and *Acomys* (E) or both (F) (error bars± SEM).

Supplemental Figure 2: **Davenport et al.**

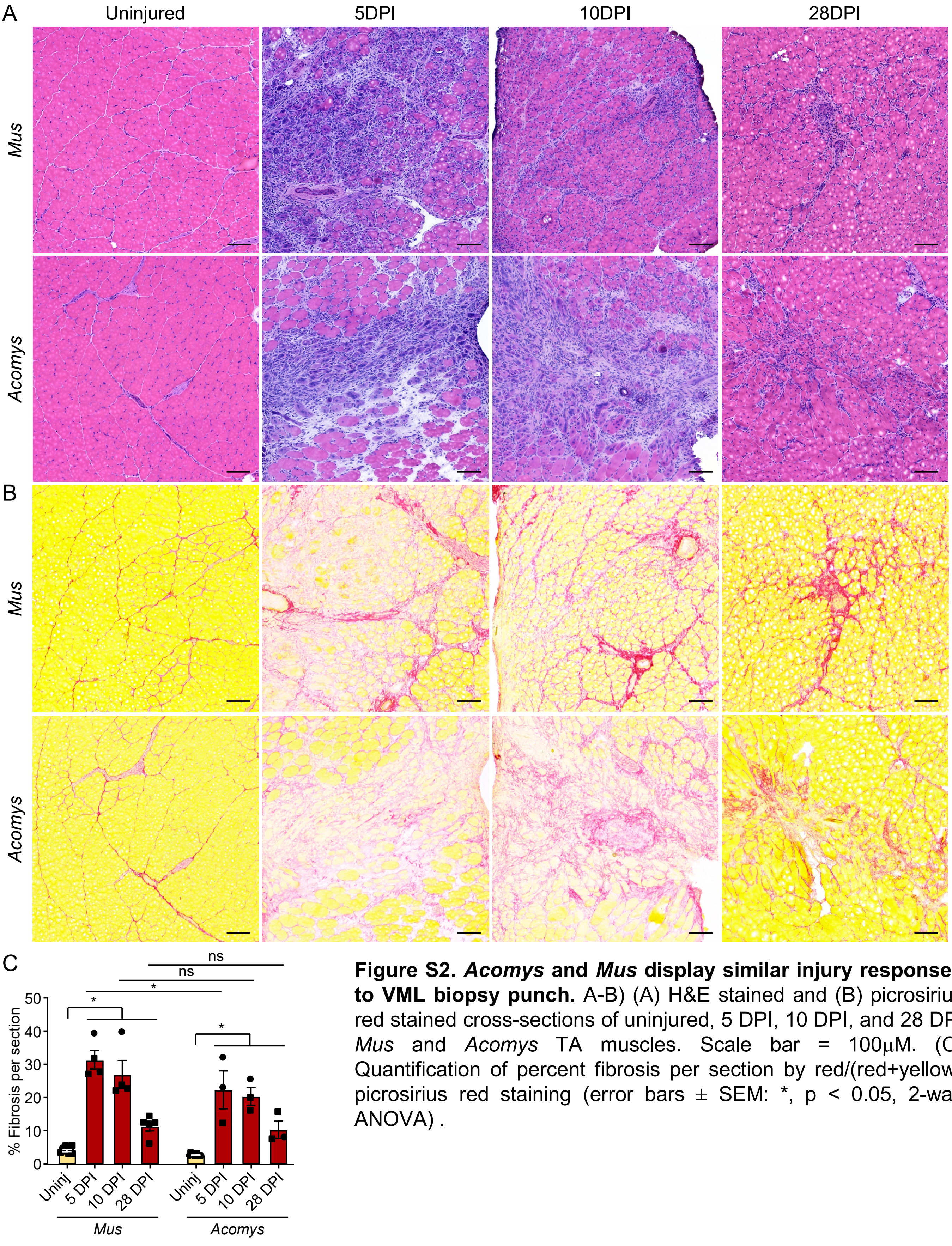

Figure S2. *Acomys* and *Mus* display similar injury responses to VML biopsy punch. A-B) (A) H&E stained and (B) picrosirius red stained cross-sections of uninjured, 5 DPI, 10 DPI, and 28 DPI *Mus* and *Acomys* TA muscles. Scale bar = 100μM. (C) Quantification of percent fibrosis per section by red/(red+yellow) picrosirius red staining (error bars ± SEM: *, $p < 0.05$, 2-way ANOVA) .

Supplemental Figure 3: **Davenport et al.**

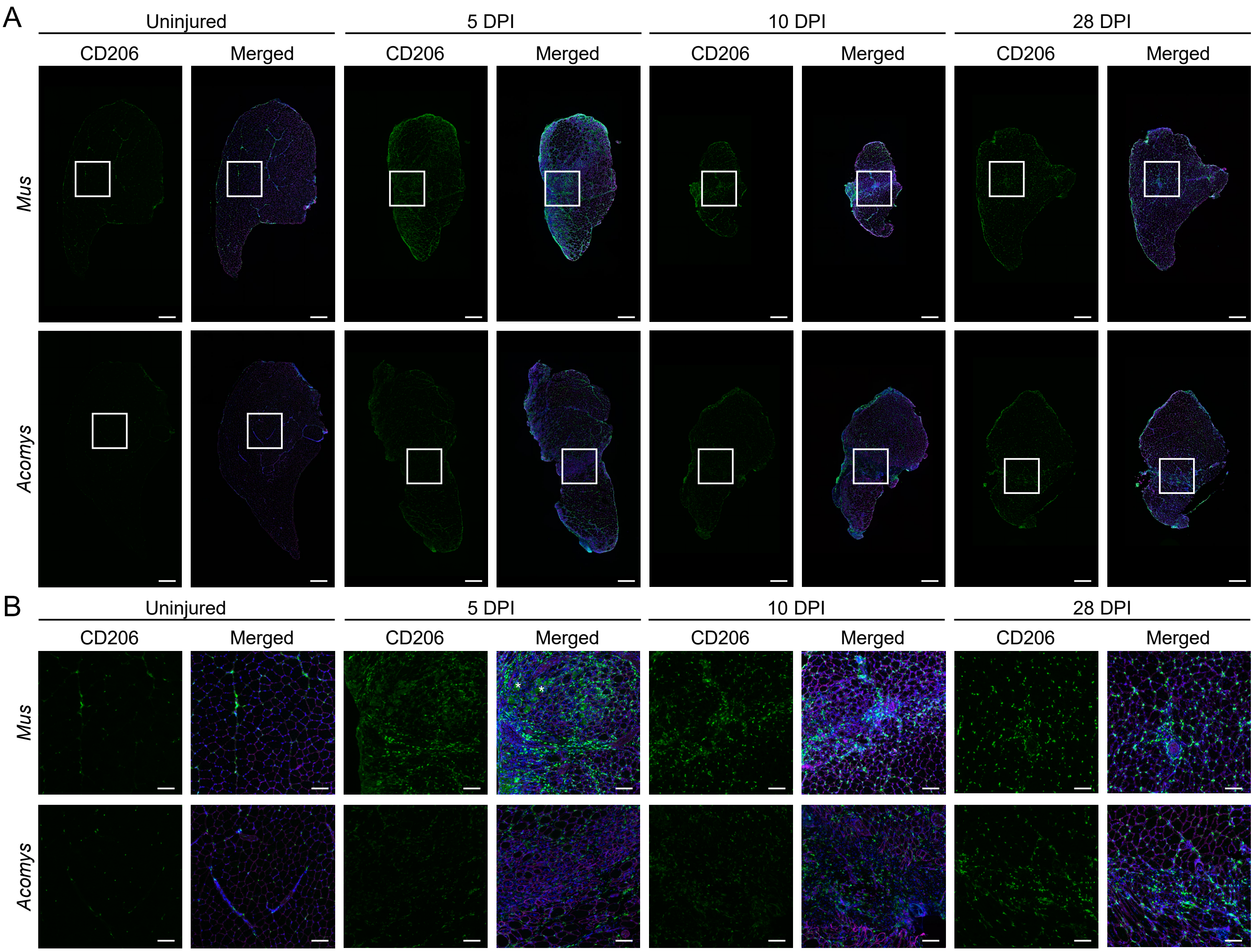

Figure S3. Macrophages across time following biopsy punch injury. A-B) Immunofluorescence of CD206, Laminin, and DAPI 5, 10, and 28 DPI in *Mus* and *Acomys*. *indicates necrotic fibers which autofluoresce. (A) Scale bar = 400µM (B) 100µM.

Supplemental Figure 4:

Davenport et al.

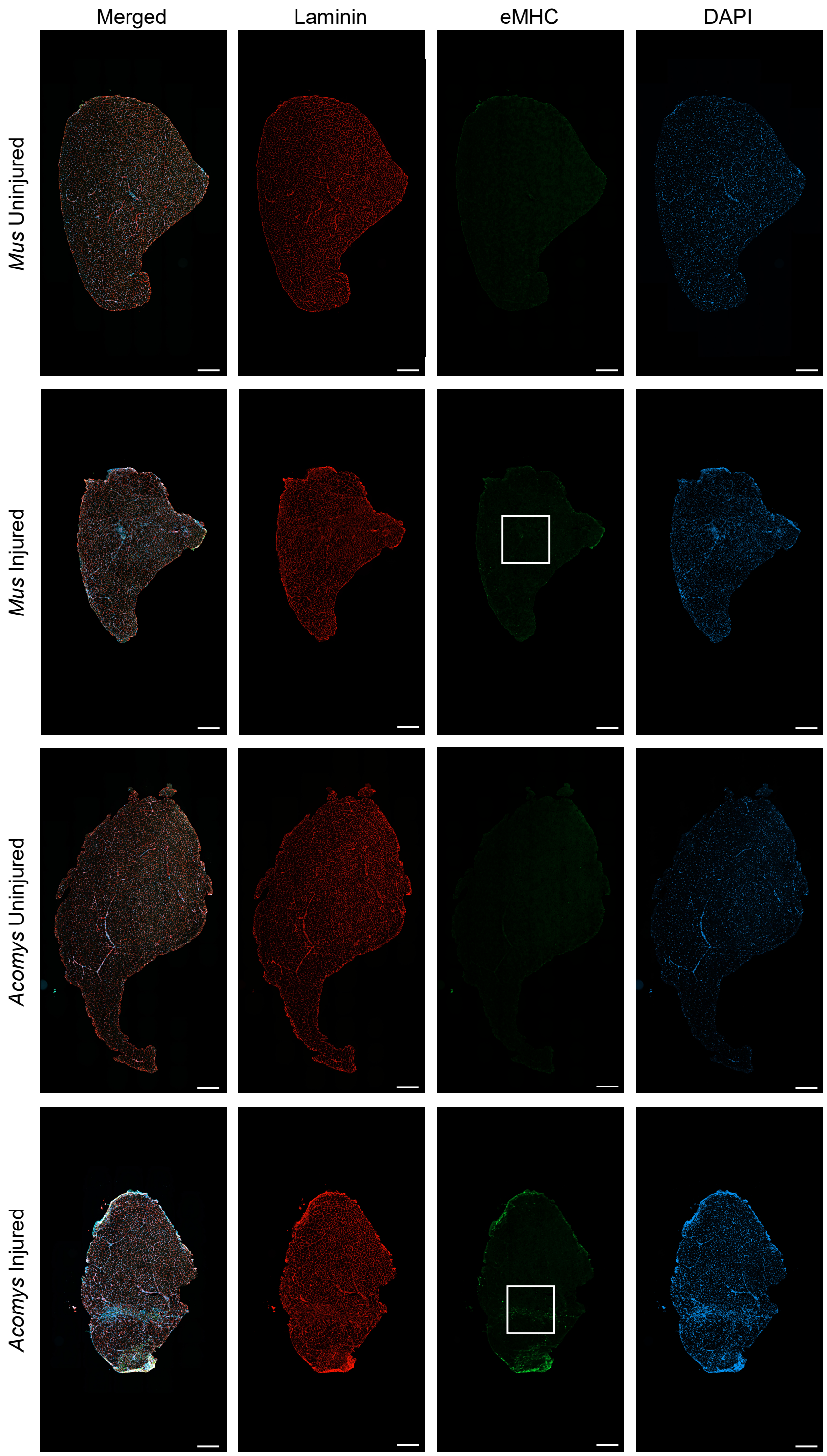

Figure S4. Full size images of biopsy punch. Immunofluorescence of eMHC, Laminin, and DAPI 28 DPI in *Mus* and *Acomys*. Scale bar = 400µM.

Supplemental Figure 5:

Davenport et al.

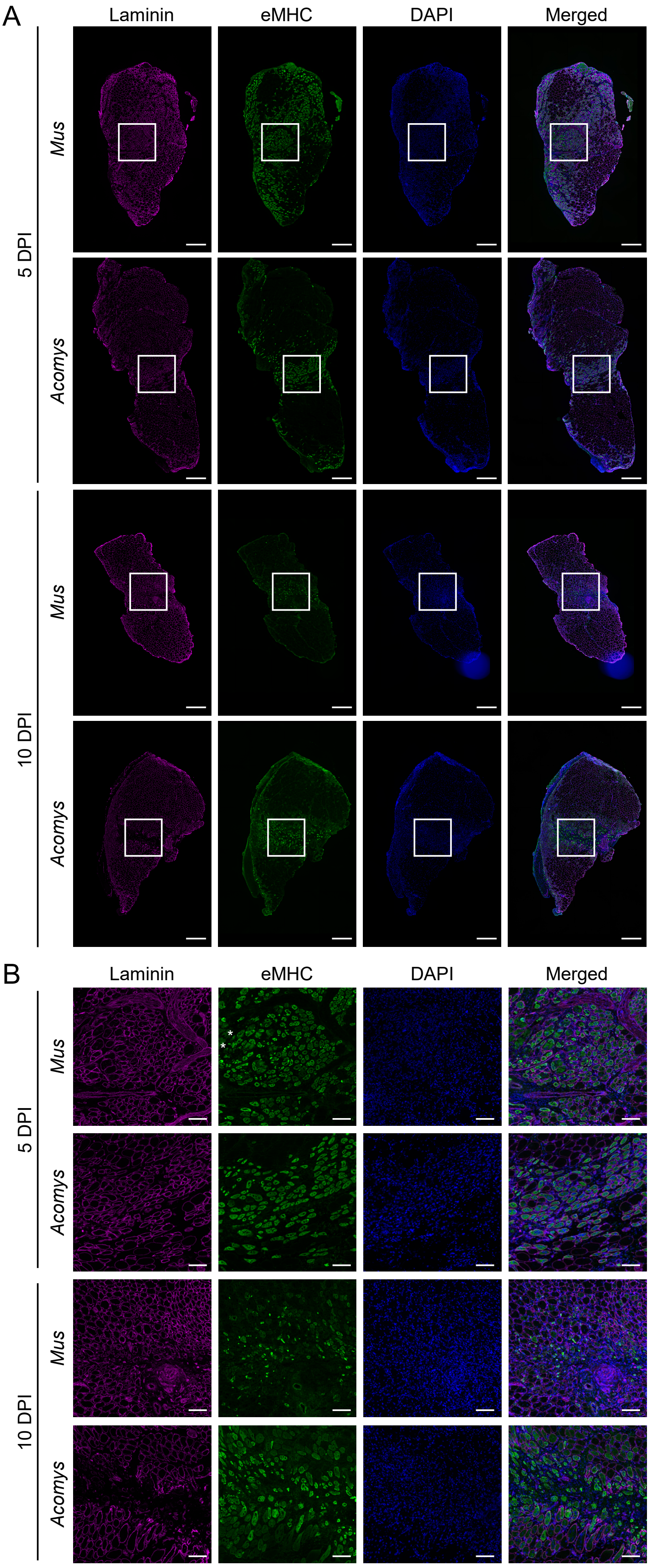

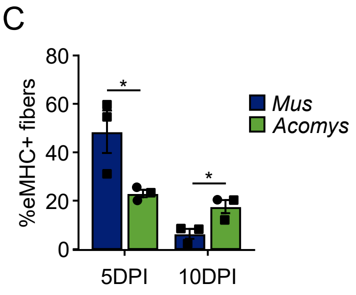

Figure S5. eMHC at early timepoints. A-B) Immunofluorescence of eMHC, Laminin, and DAPI 5 and 10 DPI in *Mus* and *Acomys*. *indicates necrotic fibers which autofluoresce. (A) Scale bar = 400μM (B) 100μM. C) Quantification of percent eMHC+ myofibers per cross-section 5 and 10 DPI in *Mus* and *Acomys* (error bars ± SEM: *, $p < 0.05$, t-test).

Supplemental Figure 6: **Davenport et al.**

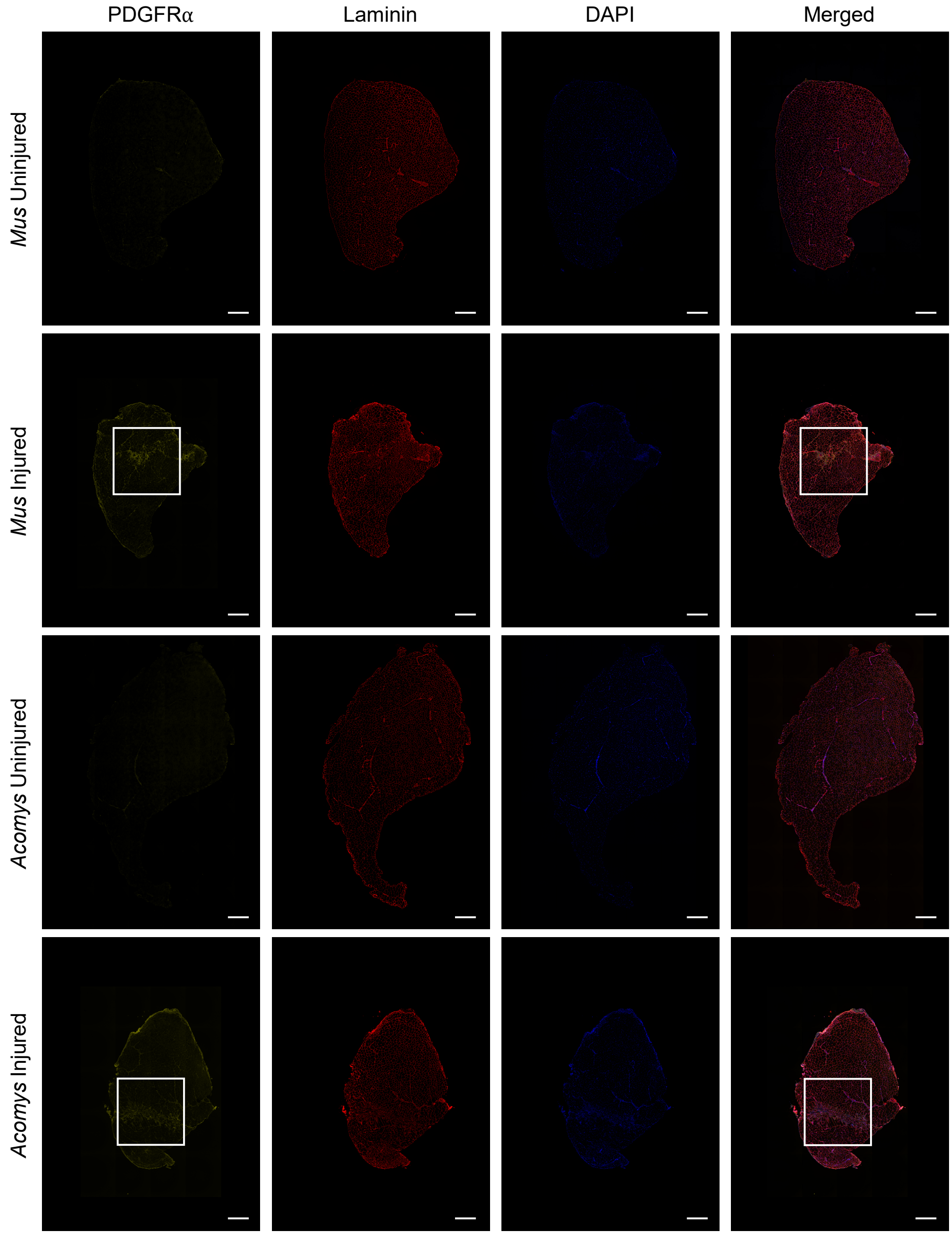

Figure S6. Full size images of biopsy punch. Immunofluorescence of PDGFRα, Laminin, and DAPI 28 DPI in *Mus* and *Acomys*. Scale bar = 400μM.

Supplemental Figure 7:

Davenport et al.

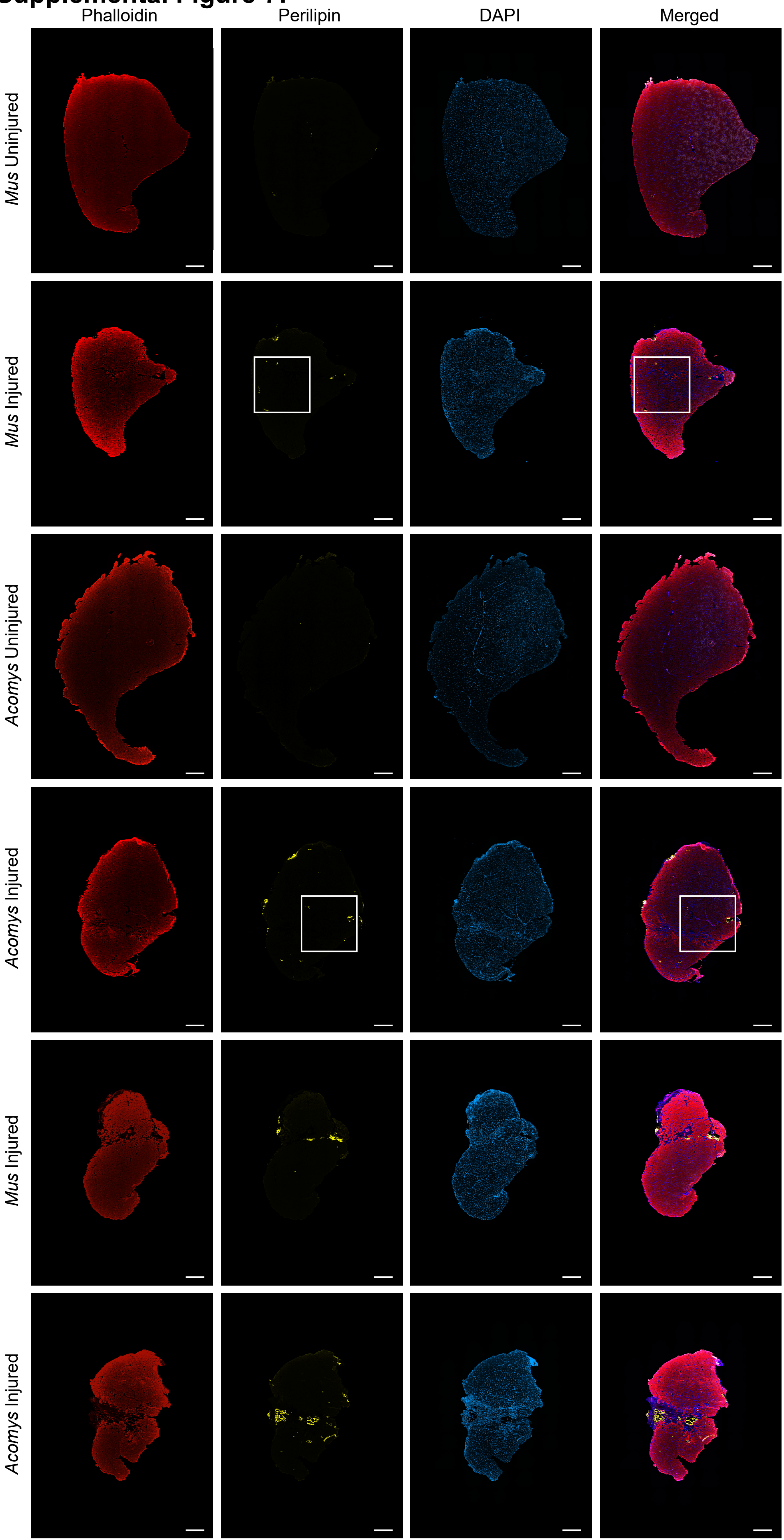

Figure S7. Full size images of biopsy punch. Immunofluorescence of phalloidin, perilipin, and DAPI 28 DPI in *Mus* and *Acomys*. Scale bar = 400μM.

Supplemental Figure 8: **Davenport et al.**

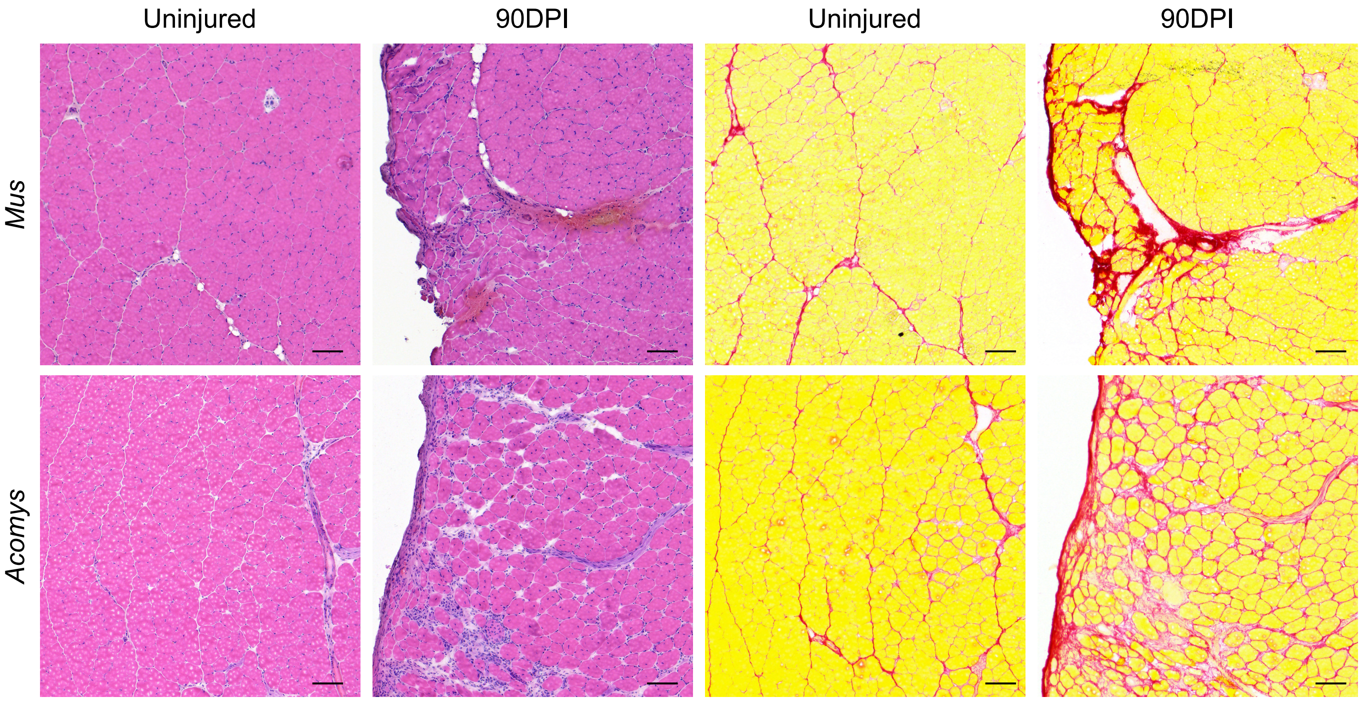

Figure S8. *Acomys* and *Mus* display similar injury responses to VML trough injury. H&E and picrosirius red stained cross-sections of *Mus* and *Acomys* TA muscles 90 days post VML trough injury. Scale bar = 100μM.

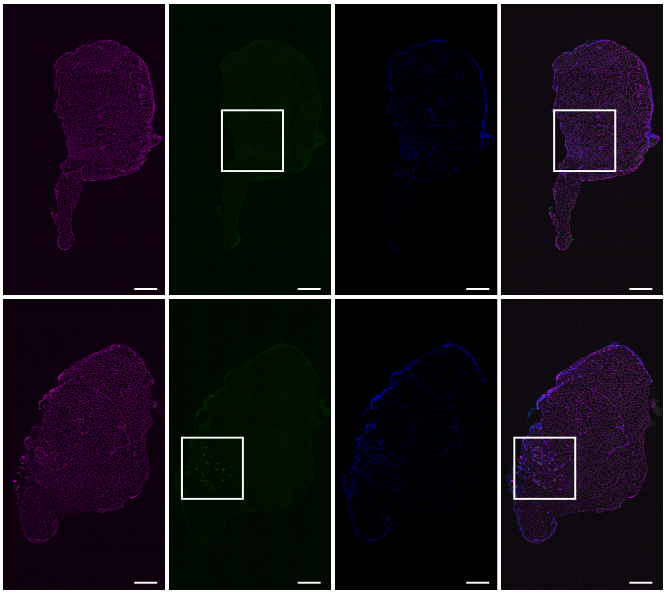

Figure S9. Full size images of trough VML. Immunofluorescence of eMHC, Laminin, and DAPI 90 DPI in *Mus* and *Acomys*. Scale bar = 400μM.

Supplemental Figure 10:

Davenport et al.

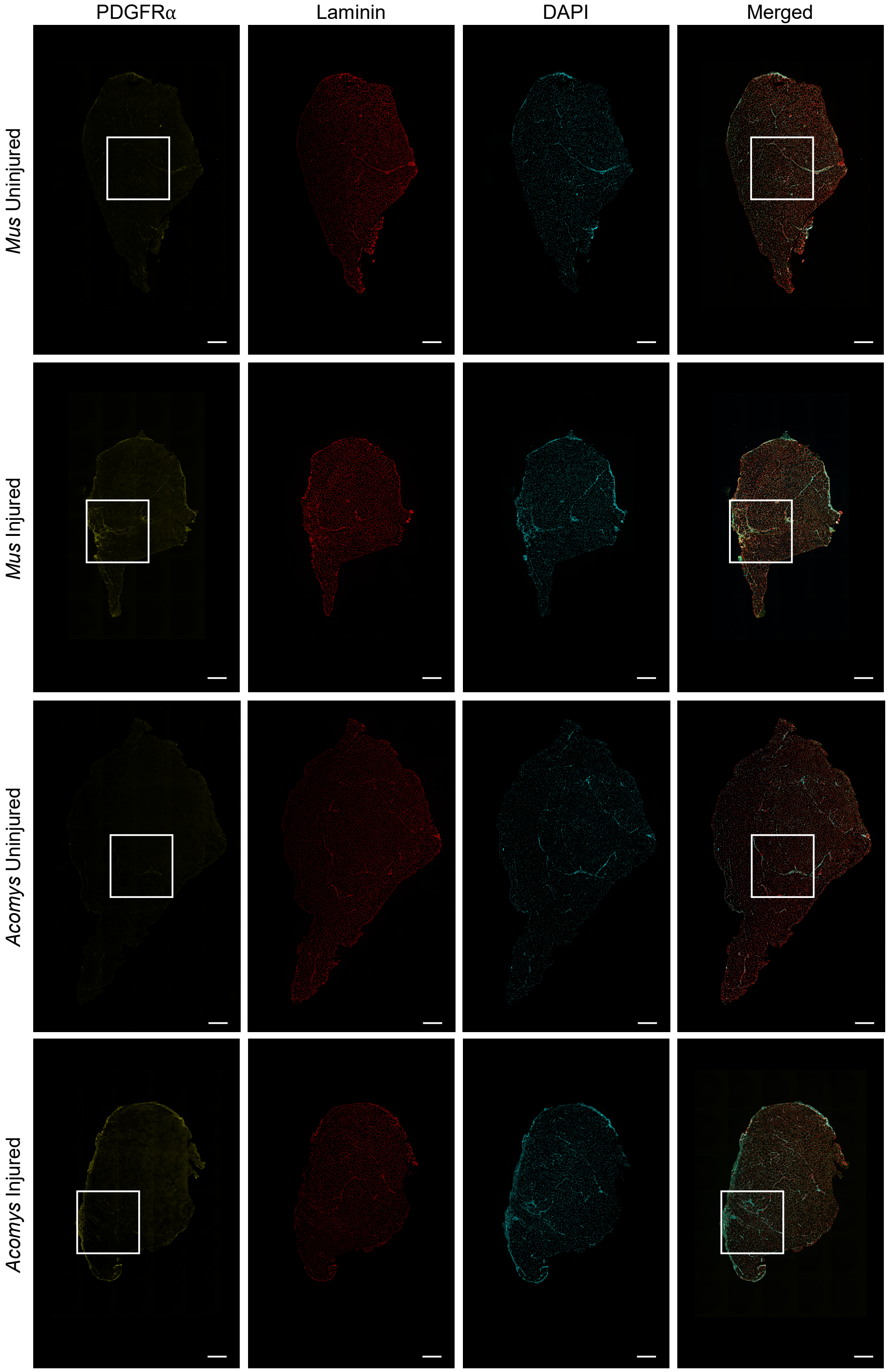

Figure S10. Full size images of trough VML. Immunofluorescence of PDGFRα, Laminin, and DAPI 90 DPI in *Mus* and *Acomys*. Scale bar = 400μM.

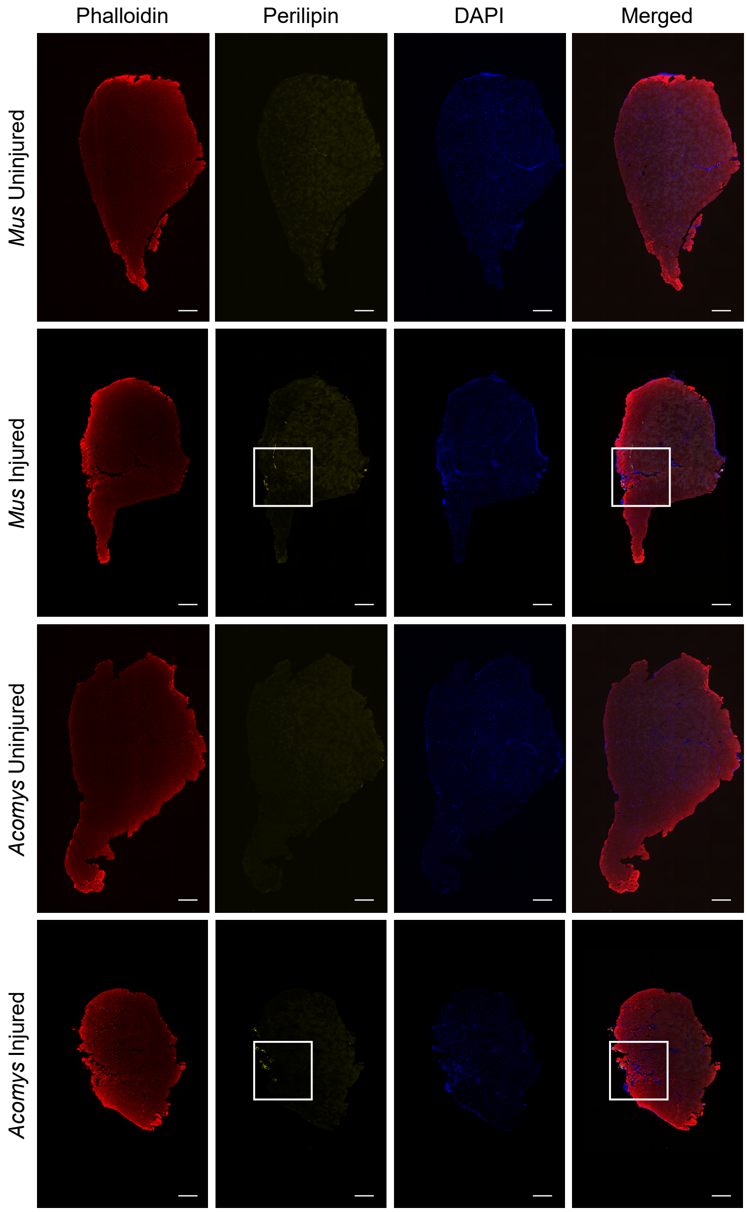

Figure S11. Full size images of trough VML. Immunofluorescence phalloidin, perilipin, and DAPI 90 DPI in *Mus* and *Acomys*. Scale bar = 400μM.

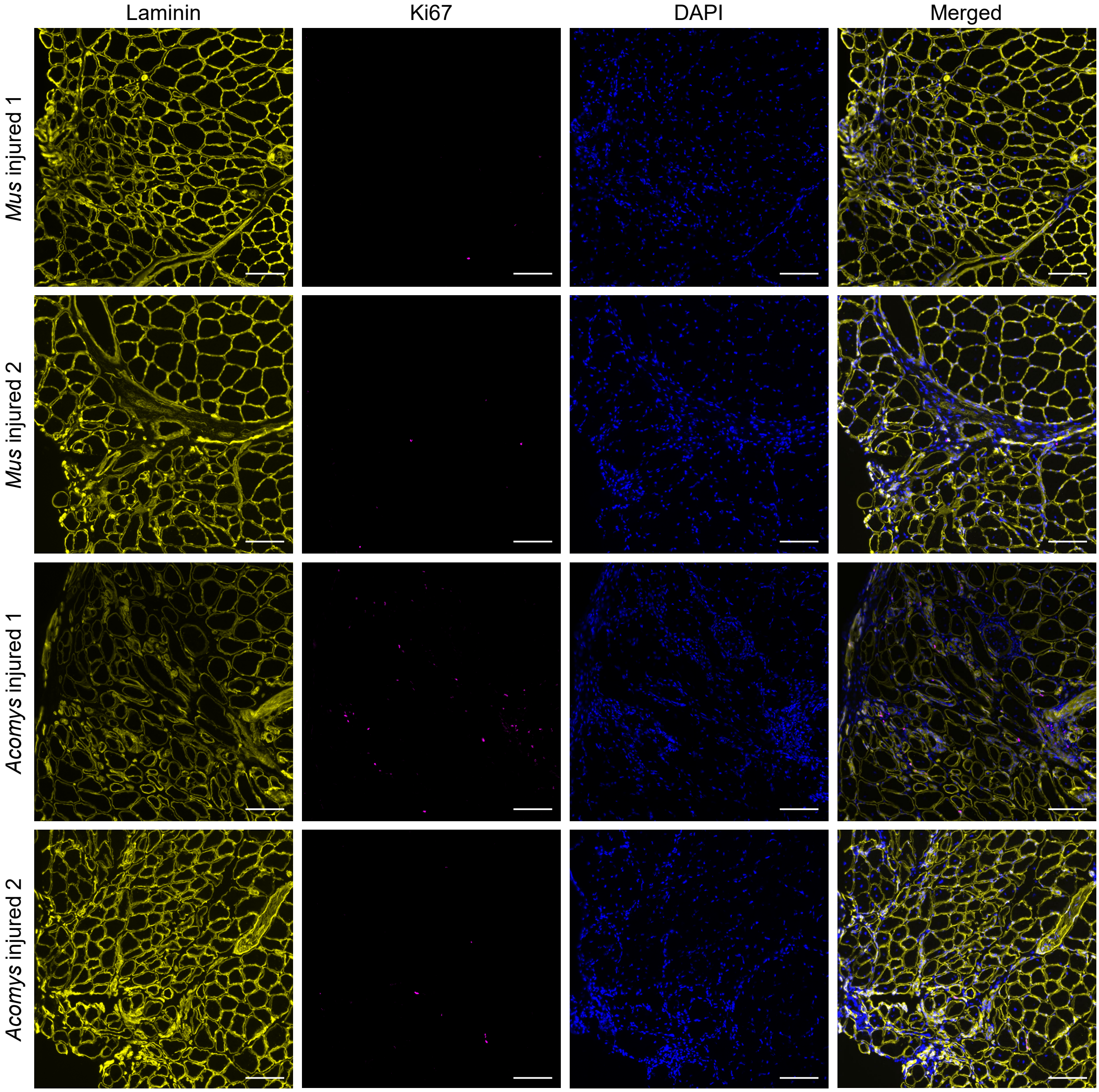

Figure S12. Proliferating cells in 90DPI trough injury. Immunofluorescence laminin, Ki67, and DAPI 90 DPI in *Mus* and *Acomys* (2 animals each). Scale bar = 100μM.
